# Trends in Cutaneous Melanoma in Nova Scotia With a Focus on 2007 to 2019

**DOI:** 10.1177/12034754241301404

**Published:** 2024-11-19

**Authors:** Rachel A. Dorey, Gordon Walsh, Ron Dewar, Peter R. Hull

**Affiliations:** 1 Faculty of Medicine, Dalhousie University, Halifax, NS, Canada; 2 Nova Scotia Health Cancer Care Program, Halifax, NS, Canada; 3 Division of Clinical Dermatology and Cutaneous Science, Department of Medicine, Dalhousie University, Halifax, NS, Canada

**Keywords:** melanoma, Nova Scotia Cancer Registry, Nova Scotia

## Abstract

**Background:**

Melanoma represents a significant public health challenge in Canada, contributing to the deaths of over 1000 individuals each year. Prince Edward Island and Nova Scotia were previously noted to have the highest incidence rates of melanoma in Canada.

**Methods:**

Data from patients diagnosed with or dying from melanoma was extracted from the Nova Scotia Cancer Registry. TNM stage was available for cases diagnosed 2007 to 2017. Incidence (1992-2019) and mortality (1992-2021) rates were examined using Join Point Trend Analysis Software.

**Results:**

Between 2007 and 2019, 2450 cases of in situ and 4063 cases of invasive melanoma were documented, of which 52.8% were male. The largest number of cases was from the 60- to 79-year age group. The most common site in females was upper limbs (in situ) and lower limbs (invasive), and for males, face, and neck (in situ), and trunk (invasive). The majority of invasive cases (71.5%) were diagnosed at stage I. Invasive melanoma incidence has been increasing by 2.7% per year since 1992, while in situ disease has increased at a greater rate (4.9% per year). The current estimate of 92% for 5 years of net survival has not changed appreciably over the same period. Survival for late-stage melanoma has shown a modest improvement for patients diagnosed over the period.

**Conclusion:**

With increasing rates of melanoma in Nova Scotia, there is a need for informed education, directed at the public and physicians, around pigmented skin lesions. This would allow the patient to detect atypical melanocytic lesions at an early stage. Sun safety practices in Nova Scotia should continue to be encouraged.

## Introduction

Melanoma represents a significant public health challenge in Canada, contributing to the deaths of over 1000 individuals each year.^1^ Between 1984 and 2019, the incidence rate for melanoma in Canada increased an average of 2.2% per year in males and 1.4% per year in females.^1^ Recent tabulations from Statistics Canada indicate that the incidence of melanoma is the highest in Nova Scotia (age-standardized incidence rate of 30.9 cases per 100,000 individuals per year in 2018).^2^ The purpose of this study was to present the descriptive epidemiology of cutaneous melanoma in Nova Scotia, including a review of the trends in incidence and mortality, the distribution of age, sex, and geographic location within the province and the clinical characteristics behaviour (in situ/invasive), anatomic location, and stage at diagnosis.

## Patient Population

As of April 1, 2023, the population of Nova Scotia was estimated to be 1,047,232.^3^ Nova Scotia residents diagnosed with a cutaneous melanoma between 1992 and 2019, or who were coded with melanoma as the cause of death between 1992 and 2021 were identified in the Nova Scotia Cancer Registry (NSCR) based on the International Classification of Diseases for Oncology (ICD-O-3) topography code C44 with histologic classification 8720 to 8790 for incidence and ICD-10 code C43 (ICD-9 code 172 for deaths prior to 2000) for mortality. For all years, data were available on Health Authority Zone of residence, sex, age, year of diagnosis, behaviour, and anatomical site. For cases diagnosed between 2007 and 2017, stage at diagnosis was also available. For the mortality analysis, age at death, sex, and year of death were available for all years. Date and cause of death were extracted to facilitate a cause-specific net survival analysis.

## Methods

Age-standardized incidence and mortality rates were calculated using the 2011 Canadian population and expressed per 100,00 population for consistency with other Canadian cancer-reporting standards.^4^ Incidence trends were examined overall and by behaviour (in situ or invasive) and sex, whereas mortality trends were examined overall and by sex. Trends were examined using JoinPoint Trend Analysis Software, for statistical analysis of trends and changes in trend.^5^ Trends are described by the Annual Percent Change. Patients with multiple distinct melanoma primaries were included based on their first diagnosis for the purposes of trend and survival analysis.^5^ Overall incidence rates and those computed by Nova Scotia Health Authority Zone included all patients’ distinct primaries. Invasive melanomas in this study were defined as melanomas at greater than stage 0. Analysis of net survival for invasive malignancies was performed in a cause-specific framework, with only deaths classified on the death certificate with melanoma as the underlying cause of death being considered death events. All other deaths are considered to be censored events. A companion analysis of net survival in a relative survival framework was performed for comparison purposes, as both methods estimate the same quantity.^6^ Survival graphs and survival proportions at the 5th year post-diagnosis were computed using SAS (version 9.4; SAS Institute Inc, Cary, NC, USA). Five years of potential follow-up was available for most patients diagnosed as late as 2017, using linkage to provincial vital statistics records.^7^ The study was approved by the Nova Scotia Health Research Ethics Board (no. 1027791).

## Results

**Figure 1.**
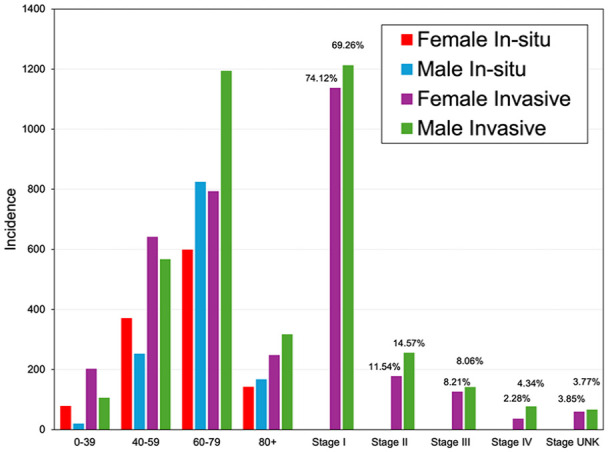
Melanoma by age and sex (2007-2019) and stage (2007-2017).

Between 2007 and 2019, there were 2450 cases of in situ melanoma and 4063 cases of invasive melanoma documented in the NSCR. For the in situ melanomas, 1188 cases were female and 1262 cases were male. For invasive melanoma, more cases were found in men than in women in 2180 cases and 1883 cases, respectively. The largest number of in situ melanoma cases were found in the 60 to 79-year age group for both females and males in 598 cases and 824 cases, respectively (Figure 1). The most common age at diagnosis for invasive melanoma for women and men was the 60 to 79 year age group in 793 and 1193 cases, respectively, with the highest number of invasive cases overall found to be at stage 1 (Figure 1).

**Figure 2.**
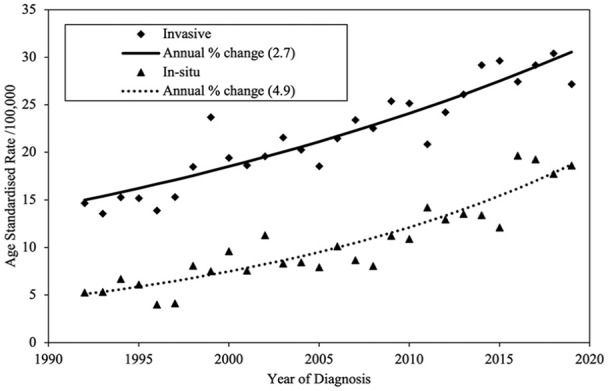
Cutaneous melanoma incidence in Nova Scotia from 1992 to 2019 (both sexes).

The long-term trends in the age-standardized incidence rate of invasive and in situ melanomas were examined over the period 1992 to 2019. In situ melanoma rates increased by 4.9% each year (4.3% in men and 5.3% in women), while invasive melanoma increased at the same rate (2.7%) in both men and women (Figure 2). This long-term increase in invasive melanoma is somewhat greater than has been observed in recently released analyses of Canadian incidence rates (1.9% per year).^8^

**Figure 3.**
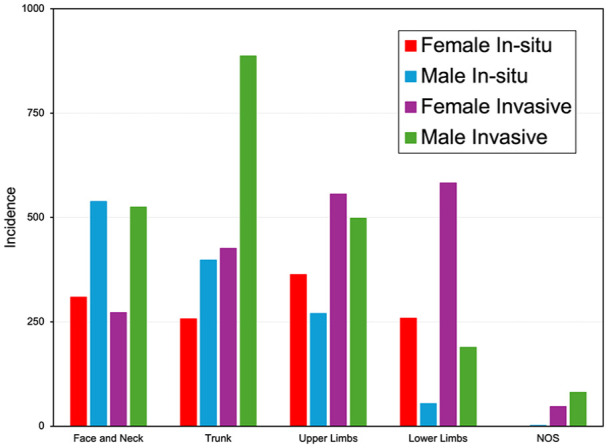
Anatomical site of melanoma by sex and behaviour from 2007 to 2019.

The most common site of in situ melanoma in females was the upper limbs in 363 cases, and for males, it was the face and neck in 538 cases. The most common site for invasive melanoma in females was on the lower limbs in 582 cases, and for males, it was the trunk in 887 cases (Figure 3). While none of the 2018 to 2019 primary melanomas have yet to be staged, about 77% of the stage 1 (AJCC 7th Edition) cases diagnosed between 2007 and 2017 had a Breslow Depth of <0.8 mm. About 20% of cases would not have undergone a sentinel lymph node biopsy and, under AJCC 8th Edition, would have been classified as stage 2.^9^

The highest number of both in situ and invasive cases were found in the most populous Central zone having the majority of dermatologists. In contrast, the Eastern zone reported the lowest age-standardized invasive incidence rate. Notably, all health zones exhibited higher rates of invasive melanoma than in situ melanoma, as illustrated in Table 1.

**Table 1.** Melanoma by Zones of Health Authority, Sex, and Behaviour Demonstrating Age-Standardized Rates From 2007 to 2019.

Health authority	Behaviour	New cases	Age-standardized rate	Lower 95% confidence limit	Upper 95% confidence limit
Nova Scotia	In situ	2450	17.3	16.6	18.0
Western		537	15.5	14.2	16.8
Northern		312	13.0	11.6	14.5
Eastern		413	15.0	13.5	16.5
Central		1188	21.5	20.2	22.7
Nova Scotia	Invasive	4063	29.3	28.4	30.3
Western		844	25.8	24.0	27.6
Northern		730	31.5	29.2	33.8
Eastern		663	24.4	22.5	26.3
Central		1826	32.8	31.3	34.3

When evaluating melanoma survival rates, we computed net survival, the probability of survival in the hypothetical world where cancer is the only possible cause of death.^6^ We evaluated the long-term trend in net survival for all invasive cases by year of diagnosis from 1992 to 2017 and were also able to evaluate stage-specific survival (stage 3 or 4) for patients diagnosed in 2007 to 2017 (Figure 1). Analysis of the trends in survival by JPSurv (JoinPoint for survival)^10^ discerned neither any significant changes over the longer time period nor any significant trend in the survival proportion for either early or late disease over the more recent period. There was a nonsignificant upward trend overall (from 55% to 57%) characterized by wide year-to-year variation.

## Discussion

Using the NSCR database, this paper describes the epidemiologic trends in cutaneous melanoma in Nova Scotia between 1992 and 2019, highlighting the variation by sex, age, anatomic site, stage, and geographic differences. While both in situ and invasive melanoma cases are increasing in Nova Scotia, there is greater concern for invasive melanomas as survival rates decrease as stage of melanoma increases.^11^

Our study highlights that melanoma incidence rates continue to increase in both sexes, with higher rates in males than in females for both in situ and invasive melanoma. This finding was also reported in previous studies using the NSCR from years 1998 to 2002.^12^ We also found that the most cases for both in situ and invasive melanomas were found in the 60- to 79-year age group, highlighting the increased risk of melanoma with age.^12^ There was an increase in the rates of invasive melanoma in Nova Scotia, 71.5% were diagnosed in stage 1, suggesting cases at the earlier in situ stage were not recognized by the patient, evolved rapidly, or there was an inordinate delay in the diagnosis of a lesion of concern. The reason for this increasing trend in melanoma in Nova Scotia is unclear. We can speculate that it relates to a founder population with lighter skin types from Scotland and Ireland, greater sun exposure from changes in clothing fashions, and more leisure time spent in the sun.

Similar to other national studies, the anatomical distribution of in situ and invasive melanomas in NS is different based on sex.^11^ The most common site in women was the upper limbs for in situ and the lower limbs for invasive melanoma, and in men, it was the face and neck for in situ and the trunk for invasive. This is in keeping with our previous study with lower extremity lesions being more likely to be invasive in female patients.^13^

When considering the survival rates of early- versus late-stage melanomas, we found that at the 5th year post-diagnosis, survival rates were higher in early-stage melanoma, which was not surprising, as the earlier the melanoma is diagnosed, the better the patient outcomes.^14^ Examination of the long-term trend in 5 years of survival for melanoma reveals very little change from 1992 to 2019. Incidence rates have increased over the same period at a rate that exceeds most other cancer types, while melanoma mortality rates have remained relatively stable over the same period. Others have seen similar patterns in US (SEER) data.^15^ This may indicate that more slowly progressive melanoma is becoming more common while those with a more aggressive growth pattern remain more constant. At this point in time, it is not possible to clinically predict which early melanomas are likely to behave in a benign manner or are destined to be aggressive and spread.

The life-extending benefit of immunotherapies is not yet appreciated. These were introduced in 2012 in Nova Scotia as monotherapies. Future reviews will be of great interest.

## Limitations

One of the limitations of our study included the inability to analyze data past 2019.

## Conclusions

This paper provides an analysis of population-based data for both in situ and invasive melanomas in Nova Scotia from 1992 to 2019 and provides some details of prognostic factors for more recently diagnosed cases. The findings of this study identified that older males in Nova Scotia had the highest risk of melanoma.

With the increasing rates of invasive melanomas in Nova Scotia, there is a need for informed education, directed at the public and physicians, around pigmented skin lesions. This would allow the patient to detect atypical melanocytic lesions at an early stage. The commonest referral to our melanoma clinic was a seborrheic keratosis. Sun safety practices in Nova Scotia should continue to be encouraged.
